# Blunt Trauma-Induced Atrial Fibrillation as First Sign of an Unspecified Cardiac Hemangioendothelioma

**DOI:** 10.1016/j.jaccas.2026.109092

**Published:** 2026-06-27

**Authors:** Stefan R. van Dinter, Hugo Maathuis, Michel W.A. Verkroost, Uta E. Flucke, Robin Nijveldt, Ad F.T.M. Verhagen

**Affiliations:** a Department of Cardiothoracic Surgery, Radboud University Medical Center, Nijmegen, the Netherlands; b Department of Primary and Community Care, Radboud University Medical Center, Nijmegen, the Netherlands; c General Practice De Linie, Doesburg, the Netherlands; d Department of Pathology, Radboud University Medical Center, Nijmegen, the Netherlands; e Department of Cardiology, University Medical Center Utrecht, Utrecht, the Netherlands

**Keywords:** atrial fibrillation, cancer, cardiac magnetic resonance, echocardiography, imaging

## Abstract

**Background:**

Hemangioendotheliomas are rare vascular tumors comprising different entities, and cardiac localization has been seldom reported.

**Case Summary:**

A 40-year-old man presented with atrial fibrillation after blunt chest trauma. Subsequent transthoracic echocardiography revealed a large right atrial mass. Cardiac magnetic resonance imaging and positron emission tomography-computed tomography showed a nonmetastatic 76 × 76-mm tumor obstructing inflow and encasing the right coronary artery (RCA). Biopsy suggested a mesenchymal neoplasm. Radical resection of the right atrium and RCA was performed, with reconstruction using a bovine pericardial patch and a saphenous vein interposition graft. Histopathology confirmed a hemangioendothelioma without a definitive subtype. Recovery was uneventful, with restored cardiac function and no recurrence for 5 years.

**Discussion:**

Management according to sarcoma principles favors complete resection. Overall survival is poor, especially in patients with metastatic disease. This case is notable for trauma-associated atrial fibrillation, oncologic RCA resection, and venous graft reconstruction.

**Take-Home Messages:**

Radical resection with coronary reconstruction is feasible. Unexplained, possibly smartwatch-detected arrhythmias after trauma warrant structural evaluation.

## Visual Summary

**Figure:**
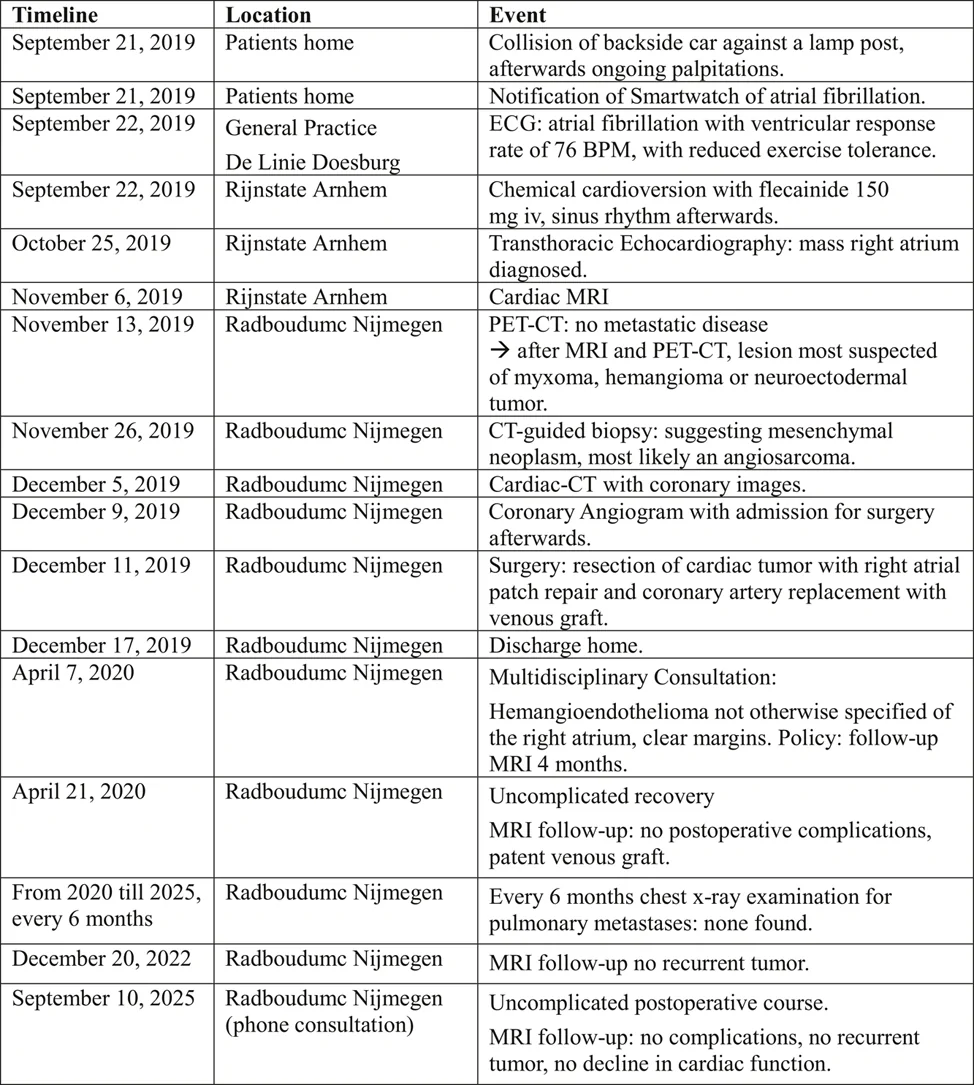

## Background

Hemangioendotheliomas are rare vascular tumors with an incidence of 0.4 cases per million person-years, comprising different entities, in the United States.^1^ We report a case of a primary, cardiac localized hemangioendothelioma not otherwise specified, which is extremely rare and not among the commonly reported primary sites.^2^

**Take-Home Messages**

• Radical resection with coronary reconstruction is feasible.
• Unexplained, possibly smartwatch-detected arrhythmias after trauma warrant structural evaluation.

## Case Presentation

A 40-year-old man with no relevant past medical or surgical history presented to the emergency cardiac care with new-onset atrial fibrillation (AF). After a car crash accident backward against a lamppost, his smartwatch (Apple Watch Series 3) detected AF. As a primary care physician, he performed an electrocardiogram at his own practice, confirming AF without a rapid ventricular response. His only symptom was mildly reduced exercise tolerance. He went to a local hospital, where pharmacologic cardioversion was successfully performed using 150 mg of flecainide intravenously. One month later, a routinely scheduled transthoracic echocardiography revealed a large right atrial mass that compromised cardiac inflow. The patient was then referred to a tertiary care center for additional imaging and urgent consultation with the cardiothoracic surgery team.

## Investigations and Differential Diagnosis

A subsequent cardiac magnetic resonance imaging (MRI) demonstrated a sharply delineated, noninvasively growing solitary 76 × 76-mm mass. The lesion showed high signal intensity on T2-weighted imaging and intermediate signal on T1-weighted turbo spin-echo sequences, with inhomogeneous perfusion and patchy, irregular late gadolinium enhancement. There was no pericardial effusion. The mass caused compression-related reduction in right atrial and ventricular function with inflow restriction to the right atrium and was noted to encase the right coronary artery (RCA). Positron emission tomography-computed tomography showed a mildly heterogeneous positron emission tomography–avid mass most likely situated intrapericardially. Because no lymphadenopathy, metastases, or pericardial effusion was observed, a differential diagnosis of a primary cardiac tumor, such as a myxoma cordis, hemangioma, or neuroectodermal tumor, was made. A computed tomography (CT)–guided diagnostic puncture was performed, and pathologic examination suggested a vascular neoplasm, most likely malignant. Given the patient's good condition and the histologic characteristics of the tumor, which appeared resectable, neoadjuvant chemotherapy was deemed unfavorable, and the patient was scheduled for urgent surgery. Preoperative cardiac CT, MRI, and coronary angiography confirmed no obstructive coronary disease but showed encasement of the RCA extending over 4 cm, with extensive vascularization toward the tumor (Video 1, for MRI and coronary angiography).

## Surgical Management

**Figure 1:**
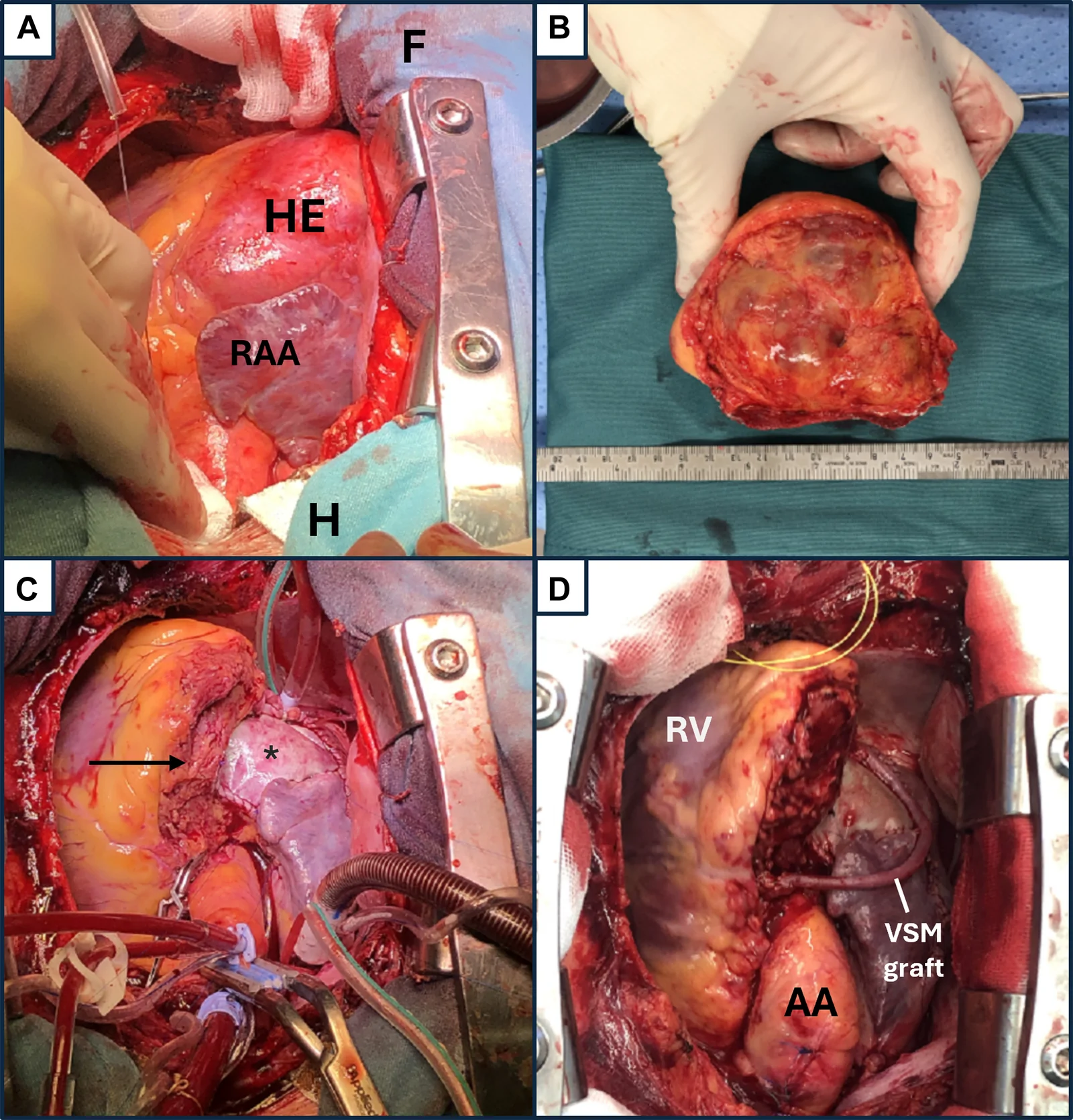
The Intraoperative Findings and Excision of the Tumor Localized in the Right Atrium With Consequent Reconstruction (A) Exposure of the heart with suspected hemangioendothelioma (HE) after sternotomy. (B) Excised tumor of ±10 × 10 cm in size. (C) The heart after excision of the tumor (including part of the right atrium and right coronary artery), with the right atrium reconstructed with a bovine pericardial patch indicated by an asterisk (∗). (D) Final intraoperative result after reconstructing the inferior wall blood supply with a saphenous vein (VSM) graft as an interposition graft. AA = ascending aorta; F = forceps; H = heart; RAA = right atrial appendage; RV = right ventricle.

A sternotomy was performed, and the pericardium was opened. The right atrium including the protruding tumor was freely mobile and nonadhesive. Extracorporeal circulation was established with arterial cannulation of the ascending aorta and venous cannulation of both the right femoral and the superior caval vein for complete cardiac bypass. No additional precautions were taken to prevent embolism, apart from minimizing tumor manipulation before cross-clamping. After achieving cardiac arrest with antegrade cardioplegia, the tumor, including the right atrial free wall and RCA, was resected, with meticulous dissection in the atrioventricular groove to preserve the right ventricular wall and tricuspid valve. The right atrium was reconstructed using a bovine pericardial patch, and a saphenous vein was used as an interposition graft in the RCA (Figure 1). The specimen was sent for pathologic examination.

## Pathological Characteristics

Microscopic findings showed a monomorphic vasoformative tumor composed of atypical cells within a sclerotic matrix, suggesting classification as a hemangioendothelioma. This was supported by immunohistochemical staining positive for CD31 and negative for CD34. Fusion gene analyses (Archer and ribonucleic acid sequencing) did not identify any fusion genes. Epigenetic analysis did not allow further classification and did not support a diagnosis of angiosarcoma. Surgical margins were negative, with the closest margin measuring approximately 1 mm. The narrowest margin was located within the atrioventricular groove toward the tricuspid valve annulus, corresponding to the tangential resection plane separating the tumor from the base of the right ventricular free wall. Cytologic analysis of the pericardial effusion showed no signs of malignancy.

## Outcome and Follow-Up

The postoperative course was uncomplicated. The patient was transferred from the intensive care unit to the clinical ward on the first postoperative day and discharged home 6 days after surgery. AF did not recur with a daily 50 mg oral metoprolol dose. Aspirin was administered to prevent occlusion of the venous interposition graft. As of today, the patient is doing well without physical limitations. A follow-up cardiac MRI shows good left and right ventricular function, no tricuspid insufficiency or signs of tumor recurrence, and a patent venous graft (Video 1).

## Discussion

Cardiac hemangioendotheliomas are exceptionally rare tumors within an already uncommon group of primary cardiac tumors. Although the overall incidence of hemangioendotheliomas across all anatomical sites is estimated at approximately 0.4 per million person-years, the heart is not among the commonly affected primary locations.^2^ Primary cardiac malignancies themselves occur in only 0.001% to 0.030% of autopsies, predominantly sarcomas.^3^ Within this context, a cardiac hemangioendothelioma is reported almost exclusively in isolated case reports, highlighting the limited evidence and the absence of disease-specific guidelines. In our case, subtyping was not successful because of the absence of a fusion gene.

Current management guidance can therefore be drawn from guidelines such as the 2022 US National Comprehensive Cancer Network sarcoma guidelines and the 2021 European Society for Medical Oncology consensus statements, as well as general principles of cardiac tumor surgery.^3^, ^4^, ^5^, ^6^ These guidelines recommend complete surgical resection (R0) as the cornerstone of treatment for localized disease when technically feasible. In our case, the decision for surgery without neoadjuvant therapy was consistent with these principles, given the absence of metastases and the resectability of the lesion. However, the cardiac location introduces unique constraints, including limited ability to achieve wide margins and the need for complex reconstruction.

The prognosis of a hemangioendothelioma is highly dependent on its subtype, which could not be determined in our case despite extensive histopathologic ribonucleic acid and epigenetic analysis. In general, hemangioendotheliomas demonstrate a heterogeneous clinical course, ranging from indolent to aggressive. Reported 5-year survival varies widely, ranging from 80% for regional disease to 46% for metastatic disease, which occurs in 55% to 65% of patients.^2^^,^^7^ Importantly, outcomes for primary cardiac malignancies overall remain poor, with median survival often less than 1 year, underscoring the potential benefit of complete surgical resection when achievable.^8^

A notable aspect of this case is the involvement and encasement of the RCA. Although several reports have described coronary artery encasement by cardiac tumors, most advocate tumor debulking with preservation of the native coronary artery rather than en bloc resection. In contrast, we performed a radical resection including the RCA, with reconstruction using a saphenous vein graft as an interposition graft. Although venous interposition grafting has been described in nononcologic settings such as aneurysmal RCA disease, its use in oncologic cardiac resection is scarcely reported.^9^ This approach allowed for oncologic complete resection while maintaining myocardial perfusion and function at 5-year follow-up and may represent a viable strategy in selected cases where coronary involvement precludes coronary preservation.

Another unique feature is the presentation with AF after blunt chest trauma. Although causality cannot be definitively established, the temporal relationship suggests that the car collision may have triggered arrhythmia in the presence of a structurally compromised right atrium. This resembles the mechanism of commotio cordis, in which mechanical impact induces electrical instability. In this case, the additional tumor mass could have increased momentum (mass × velocity) toward the right atrium, presumably leading to greater electrical instability and the induction of AF. The absence of any other preoperative and postoperative AF episodes, according to smartwatch data from 2017 to the present, supports this hypothesis. Although AF is not a typical presentation of cardiac tumors, this case highlights the importance of further evaluation following unexplained arrhythmias, particularly when associated with trauma.

The role of preoperative histologic diagnosis in cardiac tumors remains controversial. In the current case, a CT-guided biopsy suggested a mesenchymal neoplasm but did not yield a definitive diagnosis. Although a biopsy can aid in treatment planning, it carries risks including bleeding (possibly leading to tamponade), tumor seeding, and delay of definitive therapy. In addition, biopsy may increase the risk of tumor embolization into the pulmonary circulation or progressive intracardiac obstruction. Given that surgical resection would have been indicated regardless of the subtype in this case, one may question whether a preoperative biopsy can alter management in a primary resectable tumor or even whether direct-to-surgery should be the preferred strategy in selected patients to minimize embolic risk. This emphasizes the need for individualized decision-making, balancing diagnostic certainty and the need for induction therapy against the risks of biopsy and surgical delay.

Postoperative surveillance is another area lacking standardized guidance. Given the uncertain biological behavior and risk of recurrence, especially in incompletely characterized subtypes, we advocate structured follow-up with serial imaging. In line with sarcoma practice, initial cardiac MRI at regular intervals (eg, every 3-6 months in the first years) appears reasonable, although the optimal duration for specific cardiac hemangioendotheliomas remains undefined.^10^

More broadly, this case highlights several recurring challenges in rare cardiac tumors: diagnostic uncertainty, a lack of standardized treatment pathways, and the need for multidisciplinary management. Referral to specialized centers with expertise in both cardiac surgery and sarcoma care is essential to optimize outcomes.

## Conclusions

A cardiac hemangioendothelioma is a rare tumor lacking dedicated guideline-based management. This case demonstrates that radical surgical resection and reconstruction of a cardiac hemangioendothelioma invading the right atrioventricular groove can be feasible under sound oncologic principles, resulting in long-term event-free survival and excellent functional outcomes. In the absence of clear subtype classification and evidence-based guidelines, management should be guided by general sarcoma principles, multidisciplinary evaluation, and individualized risk assessment, with structured postoperative surveillance given the uncertain risk of recurrence. This case further highlights the importance of thorough evaluation of atypical cardiac arrhythmia presentations such as blunt trauma-induced AF.

## Funding Support and Author Disclosures

Dr Maathuis contributed as a patient-author to this paper. All other authors have reported that they have no relationships relevant to the contents of this paper to disclose.

## Appendix

Video 1 Video Showing the Preoperative Imaging of the Cardiac Tumor Hemangioendothelioma and Its Localization in the Right Atrium with Encasement of the Right Coronary Artery, Consisting of Preoperative Cardiac Magnetic Resonance Imaging and Coronary Angiography and Postoperative Imaging with Magnetic Resonance Imaging
